# Antibiotic-Loaded Hydrogel, Calcium Bead, and Cement in Periprosthetic Joint Infection Following Hip-Knee Arthroplasty: A Systematic Review and Meta-Analysis of Commercially Available Products

**DOI:** 10.5435/JAAOSGlobal-D-25-00304

**Published:** 2026-10-14

**Authors:** Robin Novriansyah, Danendra Rakha Putra Respati, Zahra Fathin Sadjidah, Davendra Putra Aryasatya, Maritza Bintang Rismadha, Fitri Mutmainnah, Kevin Christian Tjandra, Nur Farhanah

**Affiliations:** From the Departement of Surgery, Faculty of Medicine, Universitas Diponegoro, Semarang, Indonesia (Novriansyah); the Faculty of Medicine, Universitas Diponegoro, Semarang, Indonesia (Putra Respati, Sadjidah, Aryasatya, Rismadha, and Tjandra); the Faculty of Medicine, Universitas Andalas, Padang, Indonesia (Mutmainnah); the Division of Tropical Medicine and Infectious Diseases, Department of Internal Medicine, Faculty of Medicine, Universitas Diponegoro, Semarang, Indonesia (Farhanah); and the Dr. Kariadi General Hospital, Semarang, Indonesia (Novriansyah and Farhanah).

## Abstract

**Background:**

Local antibiotic delivery systems—including defensive antibacterial coating (DAC), calcium sulfate beads (CSBs), and antibiotic-loaded bone cement (BC)—have been developed to prevent and manage periprosthetic joint infection (PJI). However, their comparative effectiveness remains unclear.

**Methods:**

A systematic search was conducted across PubMed, Cochrane, SpringerLink, ProQuest, and Scopus. Eligible studies involved adults undergoing total hip arthroplasty or total knee arthroplasty and evaluated DAC, CSB, or BC. Inclusion criteria encompassed randomized controlled trials, cohort studies, or matched case-control studies with control groups and relevant outcomes. Key endpoints included infection rate, C-reactive protein, Knee Society Score, Harris Hip Score, and length of hospital stay (LoS). Meta-analyses were done using RStudio with the meta, metafor, and netmeta packages. Effect estimates were presented as odds ratio or mean differences (MDs) with 95% confidence intervals. Evidence quality was assessed using the GRADE approach.

**Results:**

Seventeen studies involving 3672 patients were included. DAC showed the greatest reduction in PJI (odds ratio = 0.14; 95% confidence interval, 0.04 to 0.42; *P* < 0.001) and a modest improvement in Harris Hip Score (MD = 2.77; *P* < 0.01), whereas CSB and BC showed less consistent benefits. DAC was also associated with reduced length of hospital stay (MD = −11.29 days; *P* < 0.05). Meta-regression identified male proportion as a moderator of C-reactive protein and intervention type as a determinant of length of hospital stay.

**Conclusion:**

DAC demonstrates superior efficacy among local antibiotic carriers in reducing PJI and enhancing postoperative outcomes in total hip arthroplasty and total knee arthroplasty.

Arthroplasty has been found to be an effective surgery with a survival rate of 88%.^1^ This procedure helps enhance well being by reducing symptoms and improving mobility and independence. Nevertheless, there are still complications to watch out for, one of them being periprosthetic joint infection (PJI).^2^ The prevalence of PJI after total hip arthroplasty (THA) or total knee arthroplasty (TKA) lies between 0.5% and 3%, but it can grow up to 5% in patients with rheumatoid arthritis, skin ulceration, diabetes, systemic lupus, poor nutrition, obese, and/or urinary tract infections.^3,4^ When PJI occurs, hospitalization will be prolonged accompanied with the use of antibiotics (AB). If PJI is not successfully managed, implant removal and new implant placement may be necessary, thus increasing mortality risk.^4^ There is also a financial burden to bear. In high-income countries, the cost of two-stage septic revision with re-revision can reach up to US$81,938, about 2.5 times higher than revisions without re-revision, and nearly double the cost of aseptic revisions. The main cost drivers are perioperative care (33%), prosthesis (28%), and hospital stay (22%).^5,6^

*Staphylococcus aureus* and *Staphylococcus epidermidis* are the two bacteria frequently associated with arthroplasty infections. They can create biofilm, which is a cluster of one or more microbial species, lodged in self-generated matrix and attached to either a living or nonliving surface. Orthopaedic implant surfaces, such as titanium and its alloys, stainless steel, cobalt-chromium, various polymeric biomaterial, and polymethylmethacrylate cement, are vulnerable to biofilm formation by bacteria.^7^ Biofilm formation can be characterized as four-phase processes: cell adhesion, aggregation, biofilm maturation, and cellular detachment, which is completed within 12 to 18 hours.^8^ Once formed, biofilms make bacteria highly resistant to both the human immune system and antibiotic treatment, making them difficult to eradicate.^9^

There have been many interventions and research done to prevent PJI after arthroplasty across the perioperative period. Routine administration of prophylactic systemic antibiotic is the usual protocol for patients receiving orthopaedic devices to prevent infections. However, this approach has several challenges, including the necessity for accurate timing of administration, insufficient drug levels at the target location, and limited efficacy in eliminating bacteria on the implant surface or within biofilms.^8^ A study by Gristina et al^10^ explained that there will be a bone regeneration and remodeling around the implant surfaces to protect it against bacterial colonization. Yet, bacteria can still infect the implant even with osteointegration or fibrous tissue encapsulation. Currently, an antibiotic-loaded material has been used to distribute high local drug concentrations directly to the infection site.^4,11,12^

Local antibiotic-delivery systems are materials that are applied directly to or around the implant site and are intended to release antimicrobial agents locally, resulting in high concentrations at the surgical interface while minimizing systemic exposure. In arthroplasty, these systems include antibiotic-loaded bone cement (BC), calcium sulfate beads (CSBs), and biodegradable hydrogel coatings.^13^ Antibiotic hydrogel is one of the interventions used during the preoperative stage to prevent PJI. One of the hydrogel often found on the market is defensive antibacterial coating (DAC). It consists of hyaluronan and poly-d,l-lactide, which are covalently bonded to form a stable composite. DAC will go through hydrolytic degradation in the body and release antibiotic activity, such as glycopeptides, aminoglycosides, and fluoroquinolones with concentrations of 2% to 10%.^4,14^

CSBs have also been used as antibiotic carriers in arthroplasty, as they are capable of releasing antibiotic concentrations exceeding the minimal inhibitory concentration required to eradicate common PJI pathogens and biofilms.^11,14^ In addition to antibiotic-loaded DAC and CSB, antibiotic-loaded BC is widely used for local antibiotic delivery by combining cement powder with antibiotics in predefined ratios to form beads or prosthetic components. Following implantation, antibiotic-loaded BC interacts with surrounding serum and inflammatory fluid, enabling gradual antibiotic release and sustained high local concentrations that enhance bacterial eradication.^15^

In this study, the primary outcome of interest was PJI, defined according to established arthroplasty criteria as a deep surgical site infection involving the joint space and/or prosthetic components. For clarity and consistency, the term “infection” is used throughout the article to refer exclusively to deep PJI, unless otherwise specified. Terms such as reinfection (infection occurring after initial eradication), early postoperative infection (infection developing within the early postoperative period), and deep infection are explicitly defined and applied consistently where reported by the original studies. This study therefore aims to compare the effectiveness of three local antibiotic delivery systems—DAC, CSB, and BC—in preventing PJI. We hypothesize that local antibiotic delivery systems differ in their efficacy for PJI prevention, with newer hydrogel-based coatings demonstrating comparable or superior effectiveness relative to standard delivery methods.

## Materials and Methods

This study was prospectively registered in the International Prospective Register of Systematic Reviews (PROSPERO) international prospective register of systematic reviews (CRD420251090485). Available from: https://www.crd.york.ac.uk/PROSPERO/view/CRD420251090485. Published July 10, 2025.

### Eligibility Criteria

The Preferred Reporting Items for Systematic Reviews and Meta-Analyses were used for this systematic review.^15^ In this study, a literature search was conducted through five databases such as PubMed, Cochrane, SpringerLink, ProQuest, and Scopus. We included randomized controlled trial (RCT), cohort studies, and matched case-control studies published in English that featured a control group and reported relevant outcomes. Studies were excluded if they were published in non-English languages, lacked full-text availability, were case reports or reviews, or involved unrelated interventions. The Population, Intervention, Comparator, and Outcome (PICO) framework was used, where the population consisted of patients with PJI following THA or TKA; the intervention involved various types of local antibiotic delivery systems; the comparison was made with other types of delivery systems; and the outcomes of interest were infection rate, C-reactive protein (CRP) levels, Knee Society Score (KSS), Harris Hip Score (HHS), and LoS.

### Data Search and Selection

To identify relevant studies, a comprehensive and systematic literature search was conducted using combined keywords and Boolean operators: “((((((hip arthroplasty) OR (knee arthroplasty)) AND (PJI)) AND (antibiotic-loaded hydrogel)) OR (DAC)) OR (CSB)) OR (antibiotic-loaded cement).” Complete, database-specific search strategies, including exact search strings, Medical Subject Headings (MeSH) terms, and applied filters, are provided in Supplementary File 2 (https://links.lww.com/JG9/A556). The search was restricted to studies published between 2015 and 2025. In PubMed, filters were applied to include full-text articles published in English within the past 10 years. In SpringerLink, ProQuest, and Scopus, filters for language and publication period were applied. For the Cochrane Library, advanced searches incorporating MeSH terms and Boolean operators were used without additional restrictions beyond the predefined time frame.

All retrieved records were imported into Mendeley Reference Manager, where duplicate entries were identified and removed automatically, followed by manual verification to ensure accuracy. Two independent reviewers (D.R.P.R. and Z.F.S.) performed the initial screening by excluding records based on study design, language, availability of full text, and duplication. Subsequent screening was conducted at the title and abstract level, followed by full-text eligibility assessment. Any discrepancies were resolved through discussion and, when necessary, adjudicated by two additional authors (R.N. and K.C.T.) to achieve consensus.

An extensive literature search was carried out in PubMed, Scopus, SpringerLink, ProQuest, and the Cochrane Library to find relevant studies published between January 2015 and March 2025. The search strategy was based on three essential ideas: (1) arthroplasty (“hip arthroplasty” or “knee arthroplasty”); (2) PJI; and (3) local antibiotic-delivery systems, such as “antibiotic-loaded hydrogel,” “DAC,” “CSBs,” and “antibiotic-loaded cement.” These terms were combined with Boolean operators (AND/OR) and modified to the unique needs of each database.

In PubMed, MeSH terms and free-text keywords were used, with filters applied for English-language, full-text articles published within the past 10 years. Similar language and publication-period filters were applied in Scopus, SpringerLink, and ProQuest. The Cochrane Library search employed advanced search techniques using MeSH terms and Boolean operators. All obtained records were inserted into Mendeley Reference Manager to remove duplicates. Two independent reviewers (D.R.P.R. and Z.F.S.) reviewed studies based on titles and abstracts, and then evaluated full-text using predetermined inclusion and exclusion criteria. Any disputes were settled through conversation, including mediation by two additional writers (R.N. and K.C.T.) to resolve.

### Data Extraction

After study selection, data were extracted into a Google Spreadsheet. Two independent authors (D.R.P.R. and K.C.T.) extracted study characteristics, including author, year, country, study design, sample size, mean age, sex distribution, subgroup classification, Newcastle-Ottawa Scale (NOS) score, and mean follow-up duration. Outcomes of interest extracted were infection rate, CRP, KSS, HHS, and length of hospital stay (LoS). When SDs were not directly reported, they were estimated from the reported median, range, and/or interquartile range using established statistical methods, assuming an approximately normal distribution. Specifically, SDs were imputed following the approach described by Wan et al and Luo et al, which provides validated formulas to estimate the mean and SD from median-based summary statistics depending on sample size.^16,17^ All imputed values and corresponding assumptions were documented, and studies requiring SD estimation are explicitly reported in Supplementary File 1 (https://links.lww.com/JG9/A555).

### Study Risk of Bias Assessment

In the study, we used the NOS from the Ottawa Hospital Research Institute to assess the cohort and cross-sectional studies risk of bias. This was done three independent authors (Z.F.S., M.B.R., and F.M.). If there were any disagreements between the three authors, conflict was discussed and solved with the validators (R.N., D.R.P.R., and N.F.). Quality appraisal was assessed based on three domains: (1) selection of participants; (2) comparability between exposed and nonexposed; and (3) outcome of ascertainment. For the NOS, each study has a maximum score ranging from 0 to 10 and articles with cohort design, score ranges from 0 to 9. Articles with a minimum score of 7 were categorized as “good” in quality, a score from 4 to 6 were categorized as “moderate”, and studies with a score below 4 were considered as “bad” in quality. Study with high risk of bias or “bad” in quality were excluded from the systematic review.

### Statistical Analysis

The results in the study characteristics table were displayed in SD and mean differences (MDs) for the patients age and follow-up duration. For the pooled analysis data on infection rate was presented as odds ratio (OR) with 95% confidence intervals (CIs). For the other outcomes, we presented the data using SD and MD and if the data were not available on the SD, we calculated it using a combination formula. Heterogeneity among studies was assessed using the I-squared (I^2^) statistic. Studies with I^2^ values greater than 50% were considered to have notable heterogeneity and were analyzed using a random-effects model (REM), whereas studies with I^2^ values of 25% or less were analyzed using a common or fixed-effects model. The meta-analysis was presented as a forest plot. We also assessed the publication bias using funnel plot analysis. If the funnel is asymmetric, and there are studies outside the funnel, there is a consideration of publication bias between the studies. Factors that may contribute to this is different methodologies between the studies.

Meta-regression, analyzed using R studio, was selectively applied to outcomes demonstrating substantial heterogeneity (I^2^ ≥ 50%) in the primary analyses, whereas outcomes with low heterogeneity were not further modeled. Results were reported as regression coefficients with 95% CI and *P* values, along with residual heterogeneity statistics and the proportional reduction in τ^2^ to describe the extent to which moderators explained between-study variability. Statistical significance was defined as *P* < 0.05, and findings were interpreted cautiously as exploratory due to the limited number of studies contributing to some models.

## Results

### Studies Characteristics

A total of 119,410 records were identified from five databases (Figure 1), with 117,084 records excluded before screening due to language, design, or lack of full text. After removing 1349 duplicates, 997 records were screened by title and abstract. Forty full-text articles were assessed for eligibility, with 23 excluded for reasons such as inappropriate intervention,^18-32^ no control group,^33,34^ different outcomes,^35-38^ and study design (protocol).^39^ In the end, 17 studies were included in the final synthesis.

**Figure 1:**
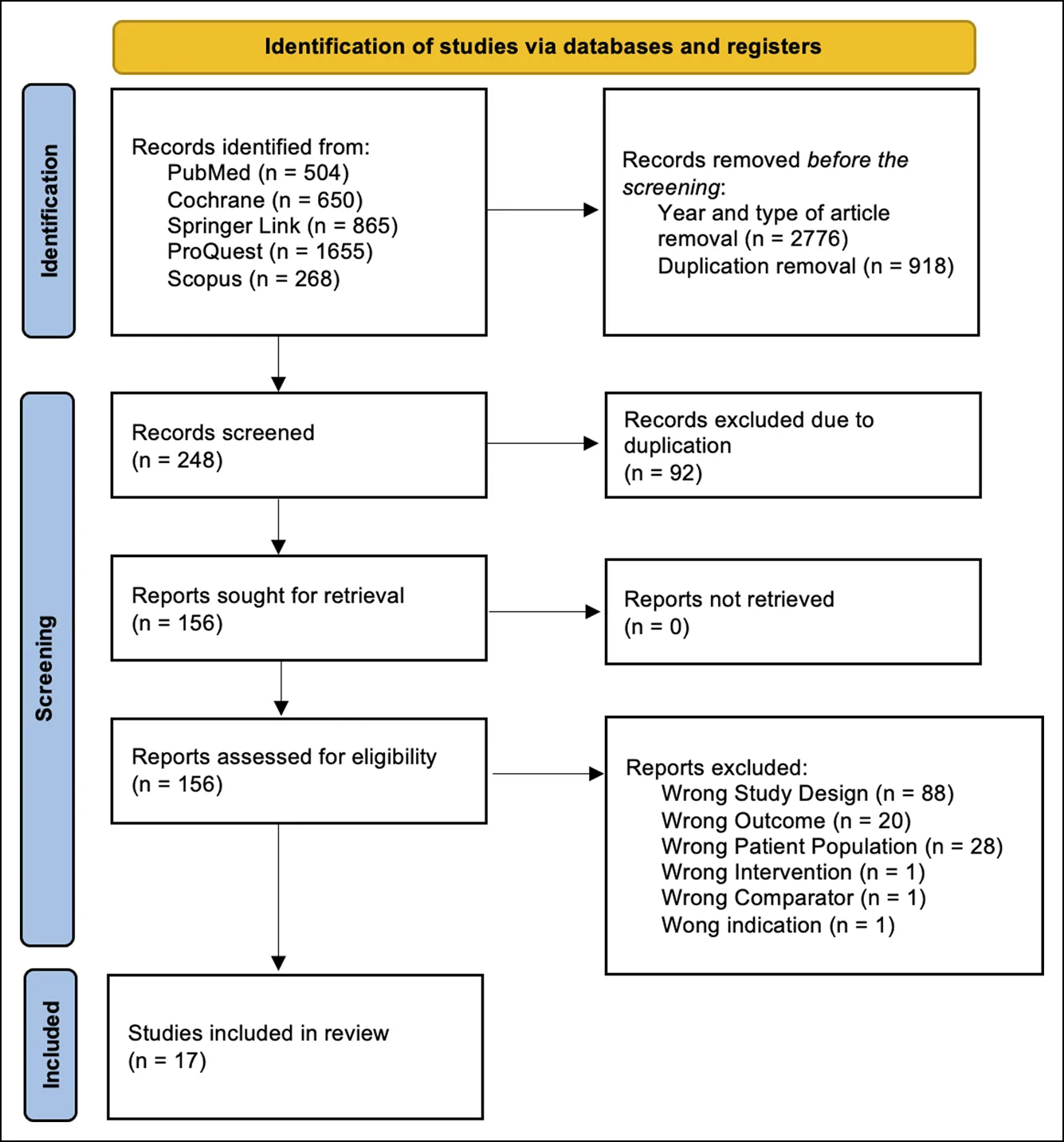
Flow chart showing PRISMA flow diagram. PRISMA = Preferred Reporting Items for Systematic Reviews and Meta-Analyses

Table 1 summarizes the characteristics and findings of 17 included studies^34,40-55^ that evaluated various local antibiotic carriers used in the management of primary and revision hip or knee arthroplasty. Six studies investigated the use of DAC hydrogel, often loaded with combinations of vancomycin (VAN), GEN, meropenem (MER), or other antibiotic. These studies consistently reported a reduction in early postoperative infections when DAC was applied during procedures such as primary arthroplasty, one- or two-stage revision, and osteosynthesis, with no observed adverse effects. DAC was frequently used with noncemented or partially cemented implants and was associated with improved infection-related outcomes, shortened antibiotic duration, and reduced LoS compared with standard topical antibiotic application.

**Table 1:** Included Studies Summary of Protocol and Findings

No	Author, Year	Population	Local AB Carrier	Treatment	Isolates	Prostheses and Cement	Type of Surgery		Main Findings
							Intervention	Control	
1	Romanò et al (2016)^55^	Primary or revision of THR/TKR	DAC, Novagenit Srl, Mezzo, Italy	DAC + VAN; DAC + GEN; DAC + VAN and MER	MRSA, MSSA	Noncemented or partially cemented implant in both group, prostheses type not reported	Primary and two-stage revision + DAC and AB	Primary and two-stage revision + topical AB	Hip or knee joint arthroplasty using DAC can reduce the rates of early postoperative site infections with no detectable adverse events or adverse effects
2	Zagra et al (2018)^40^	THR revision	DAC, Novagenit Srl, Mezzo, Italy	DAC + VAN; DAC + TEI and CEZ; DAC + VAN and RIF; DAC + VAN and MER	*S. aureus*, MRSA, streptococus	AB-loaded spacer (StageOne Zimmer or Vancogenx-space) in the first stage and noncemented prostheses with DAC in the final implants	Two-stage revision + DAC and AB	Two-stage revision + topical AB	The two-stage noncemented revision surgery's rate of reinfection may be decreased by using an antibacterial hydrogel coating without any adverse effects
3	Capuano et al (2018)^41^	THR/TKR revision	DAC, Novagenit Srl, Mezzo, Italy	DAC + VAN; DAC + VAN and MER	*S. aureus*	AB-loaded spacer (spacer G or spacer K) in the first stage and noncemented or partially cemented prostheses without DAC for control. Standard single-stage revision with DAC for intervention	Single-stage revision + DAC and AB	Two-stage revision + topical AB	Lower infection recurrence rate after one-stage exchange with DAC, compared with two-stage without coating, with reduced LoS and AB treatment duration
4	De Meo et al (2020)^34^	THR revision; femoral fracture	DAC, Novagenit Srl, Mezzo, Italy	DAC + VAN; DAC + VAN and GEN	Aseptic	Standard single-stage revision, prostheses type not reported	Single-stage revision + DAC and AB	Single-stage revision + topical AB	No PJIs were reported in the DAC group versus the six cases encountered in control
5	Zoccali et al (2021)^42^	Primary THA; oncology	DAC, Novagenit Srl, Mezzo, Italy	DAC + VAN; DAC + GEN; DAC + others	NR	Implant devices including GMRSTM, Mutars, custom-made prostheses, AB-loaded BC, bone grafts, and Trevira mesh in both groups	Primary arthroplasty/mega- prosthetic + DAC and AB	Primary arthroplasty/megaprosthetic + topical AB	The use of a AB-loaded DAC markedly reduced early surgical site infections without any reported adverse events
6	Malizos et al (2017)^43^	Primary THA/TKA; closed fracture	DAC, Novagenit Srl, Mezzo, Italy	DAC + VAN; DAC + GEN; DAC + VAN and MER	*Corynebacterium spp.*, *S. epidermidis*, *E. coli*, MRSA	Standard noncemented primary hip/knee arthroplasty, or closed fracture osteosynthesis	Primary arthroplasty/osteosynthesis + DAC and AB	Primary arthroplasty/osteosynthesis + topical AB	The AB-loaded DAC hydrogel coating markedly reduced surgical site infections without adverse effects
7	Piovan et al (2022)^44^	Acute post-TKA PJI	Stimulan, Biocomposites Ltd., Kelle, UK	CSB + VAN and GEN CSB + VAN and TOB	MRSA, MSSA, *S. epidermidis*, other gram-negative and positive	Standard DAIR procedure, no type of device or implant mentioned	Débridement; AB bead and implant retention	Débridement, AB, and implant retention	Both DAIR and DABRI showed comparable success rates (80% vs. 88%) and treatment costs, with no notable differences in infection eradication, though DABRI had a shorter follow-up period and was deemed safe
8	Mohamed et al (2019)^45^	TKA revision	Stimulan, Biocomposites Ltd., Kelle, UK	CSB + VAN and TOB	Aseptic	Standard DAIR procedure, no type of device or implant mentioned	Débridement; AB bead and implant retention	Débridement, AB, and implant retention	No notable difference in infection rates, LoS, or surgical outcomes of CSB compared with control
9	Mohamed et al (2019)^45^	THA revision	Stimulan, Biocomposites Ltd., Kelle, UK	CSB + VAN and TOB	NR	Standard DAIR procedure, no type of device or implant mentioned	Débridement; AB bead and implant retention	Débridement, AB, and implant retention	CSB showed better but no notable advantage over standard care in reducing infection rates, LoS, or surgical outcomes in aseptic revision THA
10	Sun et al (2025)^47^	TKA revision	Stimulan, Biocomposites Ltd., Kelle, UK	CSB + VAN CSB + VAN and STR CSB + VAN and MER CSB + VAN and AMP	MRSA, fungi, mycobacterium tuberculosis	Standard DAIR procedure, no type of device or implant mentioned	Débridement; AB bead and implant retention	Débridement, AB, and implant retention	CSB markedly improved infection control rates (95.7% vs. 80.9%) without increasing complications, although functional outcomes (ROM, KSS, HSS) were similar between the groups
11	Dimofte et al (2024)^48^	THR revision	Stimulan, Biocomposites Ltd., Kelle, UK	CSB + VAN and GEN	MSSA, MSCN, MRSA, MRCN, *E. coli*	Standard DAIR procedure. In two-stage revision, replacement with a spacer + AB-loaded CSB in the first stage and noncemented prostheses in the final implants	DAIR/two-stage revision + CSB with AB	DAIR/two-stage revision + topical AB	Stimulan use achieved a 93% cure rate (vs. 32% without) in DAIR cases and 100% in two-stage revisions
12	Tarity et al (2022)^49^	THR/TKR revision	Stimulan, Biocomposites Ltd., Kelle, UK	CSB + VAN CSB + VAN and TOB	MSSA, MRSA, *S. aureus*, other gram-negative	Standard DAIR procedure, no type of device or implant mentioned	Débridement; AB bead and implant retention	Débridement, AB, and implant retention	No notable difference in infection-related failure rates at 2 yr or within 90 d between DAIR procedures with and without AB-loaded CSB
13	Reinisch et al (2022)^50^	THA revision	Osteoset, Wright Medical Technology, Arlington, TN	CSB + VAN CSB + CEF	*S. aureus*, *Streptococcus spp.*, *Enterococcus spp.*, *Corynebacterium spp.*, *Propionibacterium spp.*, *E. coli*, *Pseudomonas aeruginosa*, *Citrobacter spp.*	Standard DAIR procedure, no type of device or implant mentioned	Débridement; AB bead and implant retention	Débridement, AB, and implant retention	DAIR with AB-loaded CSB markedly reduced failure rates (15% vs. 79%) and improved infection-free survival compared with standard DAIR without AB-CaSO_4_
14	Sakellariou et al (2015)^51^	TKA revision	PerOssal, Osartis GmBH, Dieburg, Germany	CSB + VAN CSB + RIF CSB + AMI	*S. epidermidis*, *S. aureus*, *Pseudomonas aeruginosa*, *Streptococcus spp.*, *Proteus mirabilis*, *Enterococcus spp.*	Replacement with a spacer + AB-loaded CSB in the first stage and noncemented prostheses in the final implants	Two-stage revision + AB-loaded HA and cement	Two-stage revision + topical AB	Combining PerOssal with cement spacers reduced CRP levels faster (*P* < 0.05) and showed a lower reinfection rate (6.67% vs. 16.13%, *P* = 0.192), although not statistically significant, compared with cement spacers alone
15	Wang et al (2015)^52^	Primary TKA	Palacos R + G, Heraeus Medical GmbH, Wehrheim, Germany	Cement + GEN	MSSA, MSCN, MRSA, MRCN	Standard primary TKA, no details on implant or procedure mentioned	Primary arthroplasty + AB-loaded BC	Noncemented primary arthroplasty	The study showed that AB-loaded BC had no effect on the prevention of deep infection after primary TKA
16	Sanz-Ruiz et al (2016)^53^	Primary THA/TKA	Palacos R + G, Heraeus Medical GmbH, Wehrheim, Germany	BC + GEN	NR	Standard primary TKA and THA, no details on implant or procedure mentioned	Primary arthroplasty + AB-loaded BC	Noncemented primary arthroplasty	AB-loaded BC has proven effective in preventing PJI following TKA and THA, while also demonstrating a favorable cost-effectiveness based on standardized cost analyses
17	Turhan et al (2018)^54^	Primary TKA	Palacos R + G, Heraeus Medical GmbH, Wehrheim, Germany	BC + GEN	Coagulase-negative *Staphylococcus*	Standard primary TKA, no details on implant or procedure mentioned	Primary arthroplasty + AB-loaded BC	Noncemented primary arthroplasty	A deep infection occurred in one patient from the AB-loaded BC group and in six patients from the control group; however, this difference was not statistically significant

AMP = ampicillin, BC = bone cement, CEF = cefuroxime, CEZ = cefazolin, CRP = C-reactive protein, CSB = calcium sulfate bead, DAC = defensive antibacterial coating, DAIR = debridement, antibiotics, and implant retention, DABRI = debridement, antibiotics, biofilm removal, and implant retention, *E. coli* = *Escherichia coli*, GMRSTM = gentamicin-loaded microsphere, HSS = Hospital for Special Surgery, HF = hydroxyapatite, KSS = Knee Society Score, MSCN = methicillin-sensitive coagulase-negative Staphylococcus, MRCN = methicillin-resistant coagulase-negative Staphylococcus, PJI = periprosthetic joint infection, RIF = rifampicin, ROM = range of motion, *S. aureus* = *Staphylococcus aureus*, *S. epidermidis* = *Staphylococcus epidermidis*, THA = total hip arthroplasty, TEI = tissue-engineered implant, THR = total hip replacement, TKA = total knee arthroplasty, TKR = total knee replacement

Eight studies examined CSB, such as Stimulan, Osteoset, and PerOssal, most commonly used during DAIR procedures or staged revisions. These carriers were loaded with various antibiotic, including VAN, tobramycin (TOB), gentamicin (GEN), streptomycin (STR), and amikacin (AMI), and were often tested against a wide range of pathogens such as methicillin-resistant Staphylococcus aureus (MRSA), methicillin-sensitive Staphylococcus aureus (MSSA), and gram-negative organisms. Several studies demonstrated markedly higher infection eradication rates with CSB use, particularly in DAIR and two-stage procedures, although some reported comparable outcomes to standard care. Three studies evaluated AB-loaded BC, primarily using Palacos R + G, preloaded with GEN, in primary TKA and THA. Although two studies showed reduced infection rates, one study reported no notable benefit of AB-loaded BC in preventing deep infections following primary arthroplasty.

### Risk of Bias

The risk-of-bias assessment and sample characteristics of the 17 included studies investigating DAC, CSB, and BC are summarized in Table 2, with the full detailed evaluation provided in Supplementary File 3 (https://links.lww.com/JG9/A557). The evidence base comprised a range of study designs, including randomized controlled trials, cohort studies, and matched case-control studies. According to the NOS, 10 studies were classified as good quality (scores 7 to 9), whereas seven studies were rated as moderate quality (scores 5 to 6). Sample sizes showed considerable variability, ranging from 15 to 2037 participants. Mean participant ages generally spanned from the mid 40s to the early 70s, and sex distribution differed across studies. Follow-up durations varied widely, from less than 12 months to more than 30 months, although several studies did not report follow-up length or age-related data.

**Table 2:** Included Studies Risk of Bias Analysis and Sample Characteristic

No	Author, Year	Design	Subgroup	NOS (S/C/O)	Sample		Mean Age		Sex		Follow-Up (mo)	
					Int	Con	Int	Con	Male	Female	Int	Con
1	Romanò et al (2016)^40^	Prospective RCT	DAC	9 (4/2/3) Good	184	189	69.0 ± 12.6	71 ± 10.6	155	218	13.3 ± 5.0	13.9 ± 2.7
2	Zagra et al (2018)^41^	Retrospective, matched case-control	DAC	7 (3/1/3) Good	27	27	63.9 ± 11.7	64.8 ± 10.1	25	29	32.4 ± 7.2	32.4 ± 7.2
3	Capuano et al (2018)^34^	Prospective, case-control	DAC	8 (4/1/3) Good	27	27	71.3 ± 13.6	71.9 ± 8.3	28	26	29.3 ± 5.0	29.2 ± 4.9
4	De Meo et al (2020)^42^	Retrospective, matched case-control	DAC	9 (4/2/3) Good	17	17	74.9 ± 11.5	75.9 ± 9.6	13	21	12.4 ± 5.7	34.3 ± 21.3
5	Zoccali et al (2021)^43^	Retrospective, matched case-control	DAC	7 (3/1/3) Good	43	43	45.6 ± 21.3	47.4 ± 19.5	48	38	24.3 ± 11.7	24.2 ± 11.5
6	Malizos et al (2017)^44^	Retrospective cohort	DAC	7 (3/2/2) Good	126	127	62.5 ± 21.2	58.6 ± 17.6	110	143	18.1 ± 5.2	18.1 ± 3.5
7	Piovan et al (2022)^45^	Retrospective cohort	CSB	8 (3/2/3) Good	17	15	64.2 ± 8.7	69.1 ± 11.4	17	15	16.1 ± 8.1	30.2 ± 13.7
8	Mohamed et al (2019)^46^	Retrospective cohort	CSB	6 (3/1/2) Moderate	49	57	64.2 ± 9.9	63.8 ± 11.9	43	63	<24	<24
9	Mohamed et al (2019)^47^	Retrospective cohort	CSB	6 (3/1/2) Moderate	48	58	62.2 ± 12.6	60.0 ± 15.3	58	48	<24	<24
10	Sun et al (2025)^48^	Retrospective cohort	CSB	9 (4/2/3) Good	47	47	65.6 ± 10.5	67.1 ± 12.3	40	54	NR	NR
11	Dimofte et al (2024)^49^	Retrospective cohort	CSB	6 (3/1/2) Moderate	45	31	66.6 ± 11.9	69.1 ± 12.2	50	26	<12	<12
12	Tarity et al (2022)^50^	Retrospective cohort	CSB	6 (3/1/2) Moderate	20	20	72.1 ± 11.8	71.1 ± 8.2	23	17	<24	<24
13	Reinisch et al (2022)^51^	Retrospective cohort	CSB	7 (3/2/2) Good	27	14	71.3 ± 14.3	65.3 ± 7.4	15	26	NR	NR
14	Sakellariou et al (2015)^52^	Retrospective cohort	CSB	6 (3/2/1) Moderate	15	31	NR	NR	8	38	35.3 ± 14.9	35.5 ± 12.6
15	Wang et al (2015)^53^	Retrospective cohort	BC	8 (3/2/3) Good	256	2037	63.3 ± 11.1	64.9 ± 10.6	389	1904	<12	<12
16	Sanz-Ruiz et al (2016)^54^	Retrospective cohort	BC	5 (2/2/1) Moderate	221	68	NR	NR	NR	NR	<24	<24
17	Turhan et al (2018)^39^	Retrospective cohort	BC	6 (2/2/2) Moderate	85	521	62.8 ± 10.3	62.8 ± 0.3	110	496	<24	<24

BC = bone cement, CSB = calcium sulfate bead, DAC = defensive antibacterial coating, NOS = Newcastle-Ottawa Scale, NR = not reported

### Reinfection Rate

Overall, antibiotic-loaded local delivery strategies were associated with a notable reduction in infection risk compared with control, with a common-effect OR of 0.22 (95% CI, 0.14 to 0.35; Z = −6.05; *P* < 0.001), indicating a strong protective effect of antibiotic-loaded interventions (Figure 2). Subgroup analyses showed notable benefits for both DAC and CSB, with the greatest effect observed in the DAC subgroup. The DAC subgroup yielded the lowest pooled effect estimate, with an OR of 0.12 (95% CI, 0.05 to 0.30; *P* < 0.001), reflecting a substantial reduction in infection risk. Similarly, the CSB subgroup demonstrated a notable protective effect with a pooled OR of 0.26 (95% CI, 0.14 to 0.49; *P* < 0.001). By contrast, the cement subgroup showed a negligible reduction in infection risk, with a pooled OR of 0.35 (95% CI, 0.14 to 0.88; *P* = 0.17), suggesting a less consistent or weaker effect compared with DAC and CSB.

**Figure 2:**
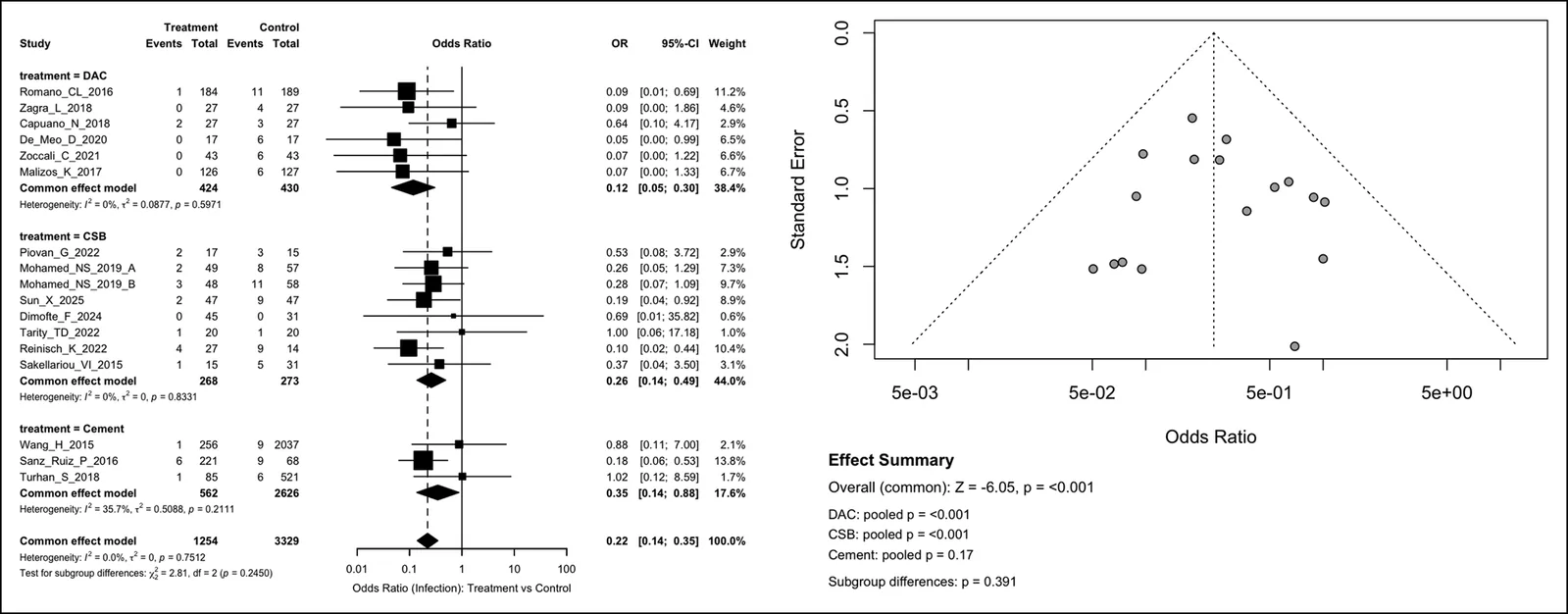
Forrest and funnel plot of DAC, CSBs, and BC effectiveness in reducing infection rate. Each outcome is expressed as an OR with a 95% CI based on a FEM. BC = bone cement, CI = confidence interval, CSB = calcium sulfate bead, DAC = defensive antibacterial coating, FEM = fixed-effects model, OR = odds ratio

Heterogeneity within subgroups was low, with I^2^ = 0% for DAC and CSB, and moderate for Cement (I^2^ = 35.7%), indicating generally consistent findings across studies. Importantly, the test for subgroup differences was not statistically significant (χ^2^ = 2.81, df = 2, *P* = 0.391), suggesting no clear evidence that the magnitude of effect differed between DAC, CSB, and cement. These results support the effectiveness of antibiotic-loaded local delivery strategies—particularly DAC and CSB—in reducing infection risk, with comparable efficacy across subgroups.

Visual inspection of the funnel plot demonstrated a relatively symmetrical distribution of studies around the pooled effect estimate, indicating a low likelihood of substantial publication bias. Studies with larger standard errors (smaller sample sizes) were dispersed on both sides of the central OR and were not confined to statistically significant regions. Several small studies were located outside the *P* < 0.05 contours, suggesting that studies with negligible or less pronounced effects were also included. Overall, the absence of marked asymmetry in the funnel plot supports the robustness and reliability of the meta-analytic findings.

### C-Reactive Protein

The forest plot (Figure 3A) presents a subgroup meta-analysis evaluating the effect of antibiotic-loaded interventions CRP levels, comparing DAC and CSB against control groups. Overall, the REM demonstrated a negligible reduction in CRP levels, with a pooled MD of −1.47 (95% CI, −3.29 to 0.35; Z = −1.58; *P* = 0.11), indicating that the intervention did not result in a statistically significant decrease in systemic inflammatory response as measured by CRP.

**Figure 3:**
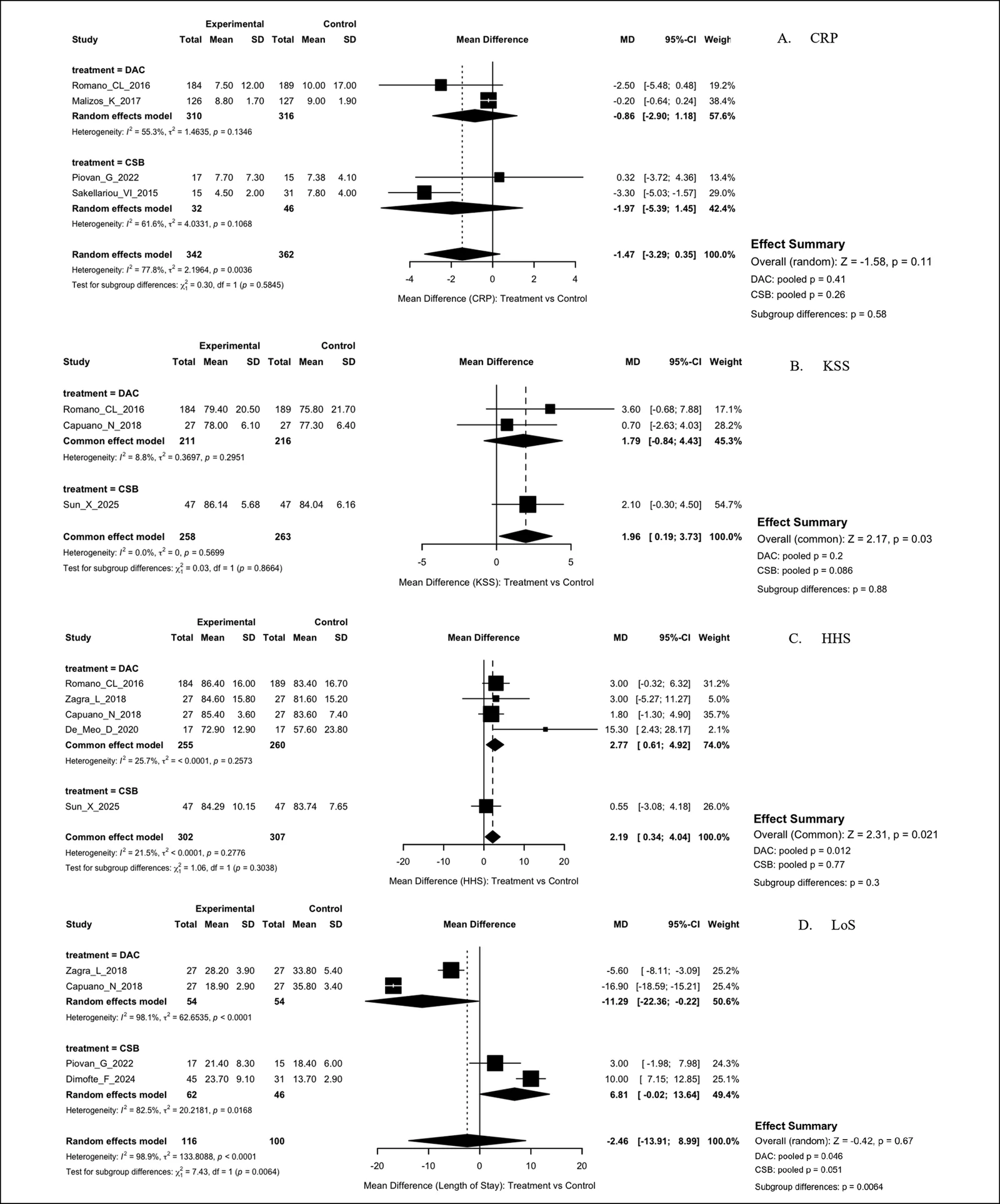
Forest plots of subgroup meta-analyses comparing DAC and CSS treatments across four outcomes. (**A**) CRP, (**B**) KSS, (**C**) HHS, and (**D**) LoS. Results are presented as MD with 95% CI using a REM and FEM. CRP = C-reactive protein, CSS = clinical success score, DAC = defensive antibacterial coating, FEM = fixed-effects model, HHS = Harris Hip Score, KSS = Knee Society Score, LoS = length of hospital stay, MD = mean difference, REM = random-effects model

In the subgroup analysis, neither intervention showed a statistically significant effect. The DAC subgroup yielded a pooled MD of −0.86 (95% CI, −2.90 to 1.18; *P* = 0.41), suggesting no clear reduction in CRP compared with control. Similarly, the CSB subgroup demonstrated a pooled MD of −1.97 (95% CI, −5.39 to 1.45; *P* = 0.26), indicating a trend toward lower CRP values, although this effect did not reach statistical significance. Heterogeneity varied across analyses. Moderate heterogeneity was observed within the DAC subgroup (I^2^ = 55.3%), whereas the CSB subgroup also showed moderate heterogeneity (I^2^ = 61.6%). The overall analysis exhibited substantial heterogeneity (I^2^ = 77.8%, *P* = 0.0036), reflecting variability in CRP outcomes across studies, potentially due to differences in study design, timing of CRP measurement, baseline inflammatory status, or perioperative protocols.

Test for subgroup differences was not statistically significant (χ^2^ = 0.30, df = 1, *P* = 0.58), indicating no evidence of a differential effect between DAC and CSB on CRP reduction. Overall, these findings suggest that although antibiotic-loaded local delivery strategies may reduce infection rates, their impact on systemic inflammatory markers such as CRP remains inconclusive, with no notable differences observed between DAC and CSB in the current evidence base.

### Knee Society Score

Antibiotic-loaded strategies were associated with a modest but statistically significant improvement in knee function overall (MD, 1.96; 95% CI, 0.19 to 3.73; *P* = 0.03), as shown in Figure 3B. Subgroup analyses showed directionally favorable effects for both DAC and CSB. In the DAC subgroup, two studies contributed to the analysis, yielding a pooled MD of 1.79 (95% CI, −0.84 to 4.43; *P* = 0.20). Although the point estimate favored DAC, the CI crossed zero, suggesting that the improvement in KSS did not reach statistical significance. Heterogeneity within this subgroup was low (I^2^ = 8.8%), indicating good consistency between the studies. The CSB subgroup, represented by a single study, demonstrated a pooled MD of 2.10 (95% CI, −0.30 to 4.50; *P* = 0.086). This result showed a trend toward improved functional outcomes with CSB compared with control; however, the effect was not statistically significant. As expected with a single study, no heterogeneity was observed (I^2^ = 0%).

Importantly, the test for subgroup differences was not statistically significant (χ^2^ = 0.03, df = 1, *P* = 0.88), indicating no evidence of a differential effect between DAC and CSB on KSS outcomes. Overall, these findings suggest that antibiotic-loaded local delivery strategies are associated with a modest but statistically significant improvement in knee function, as reflected by KSS, whereas individual subgroup effects remain directionally favorable but inconclusive due to limited power and sample size.

### Harris Hip Score

The forest plot (Figure 3C) presents a subgroup meta-analysis evaluating the effect of antibiotic-loaded interventions on HHS, comparing DAC and CSB with control groups. The common-effect model demonstrated a statistically significant improvement in HHS favoring the intervention, with a pooled MD of 2.19 (95% CI, 0.34 to 4.04; Z = 2.31; *P* = 0.021), indicating better postoperative hip function in patients receiving antibiotic-loaded strategies.

Within the DAC subgroup, four studies contributed to the analysis, yielding a pooled MD of 2.77 (95% CI, 0.61 to 4.92; *P* = 0.012), which reflects a statistically significant improvement in hip function compared with control. Heterogeneity within this subgroup was low to moderate (I^2^ = 25.7%), suggesting reasonable consistency across studies despite variations in study design and follow-up duration. By contrast, the CSB subgroup, represented by a single study, demonstrated a pooled MD of 0.55 (95% CI, −3.08 to 4.18; *P* = 0.77), indicating no statistically significant difference in HHS between the CSB and control groups. As only one study was included, heterogeneity was negligible (I^2^ = 21.5% overall).

Subgroup difference analysis did not reach statistical significance (χ^2^ = 1.06, df = 1, *P* = 0.30), indicating no definitive evidence of differential effects on HHS between DAC and CSB. Overall, the results suggest that antibiotic-loaded local delivery approaches are associated with a small yet statistically significant improvement in hip functional outcomes, largely attributable to the DAC subgroup, whereas conclusions regarding CSB remain constrained by limited available data.

### Length of Stay

The forest plot (Figure 3D) presents a subgroup meta-analysis assessing the effect of antibiotic-loaded interventions on LoS, comparing DAC and CSB with control groups. REM showed no statistically significant difference in LoS between treatment and control, with a pooled MD of −2.46 days (95% CI, −13.91 to 8.99; Z = −0.42; *P* = 0.67), indicating substantial uncertainty around the overall effect.

Two studies in the DAC subgroup demonstrated a notable reduction in LoS, yielding a pooled MD of −11.29 days (95% CI, −22.36 to −0.22; *P* = 0.046). However, heterogeneity within this subgroup was extremely high (I^2^ = 98.1%, *P* < 0.0001), suggesting considerable variability in effect sizes, likely driven by differences in patient populations, perioperative pathways, baseline infection severity, or discharge criteria. Conversely, the CSB subgroup showed a notable trend toward increased LoS, with a pooled MD of 6.81 days (95% CI, −0.02 to 13.64; *P* = 0.051). This subgroup also exhibited substantial heterogeneity (I^2^ = 82.5%, *P* = 0.0168), indicating inconsistent findings across included studies.

Importantly, the test for subgroup differences was statistically significant (χ^2^ = 7.43, df = 1, *P* = 0.0064), suggesting a true differential effect between DAC and CSB on hospital LoS. Although DAC appeared to be associated with a shorter LoS, CSB did not demonstrate a similar benefit and may be associated with prolonged hospitalization in some settings. The results indicate that the influence of antibiotic-loaded local delivery strategies on LoS is highly variable and dependent on subgroup classification. Although DAC shows a potential benefit in reducing LoS duration, the extremely high interstudy heterogeneity limits the certainty of this finding, underscoring the need for further rigorously designed studies to clarify the determinants of LoS outcomes.

### Results Summary

Table 3 summarizes the comparative effects of antibiotic-loaded delivery strategies (DAC, CSB, and BC) across infection, inflammatory, functional, and hospitalization outcomes. For infection rate, DAC demonstrated the strongest and most consistent protective effect (OR = 0.12; 95% CI, 0.05 to 0.30; *P* < 0.001; I^2^ = 0%), followed by CSB (OR = 0.26; 95% CI, 0.14 to 0.49; *P* < 0.001; I^2^ = 0%), whereas BC showed a notable reduction (OR = 0.35; 95% CI, 0.14 to 0.88; *P* = 0.17; I^2^ = 36%). Overall, antibiotic-loaded interventions markedly reduced infection risk (OR = 0.22; 95% CI, 0.14 to 0.35; *P* < 0.001; I^2^ = 0%). By contrast, CRP outcomes were inconclusive, with notable reductions observed for both DAC (MD = −0.86; *P* = 0.41; I^2^ = 55%) and CSB (MD = −1.97; *P* = 0.26; I^2^ = 62%), and a notable overall effect accompanied by substantial heterogeneity (MD = −1.47; *P* = 0.11; I^2^ = 78%).

**Table 3:** Summary of Outcomes

Outcome	DAC Mean/OR (95% CI)	*P* Value	I^2^	CSB Mean/OR (95% CI)	*P* Value	I^2^	BC Mean/OR (95% CI)	*P* Value	I^2^	Overall Mean/OR (95% CI)	*P* Value	I^2^
Infection rate	0.12 (0.05-0.30)	<0.001	0%	0.26 (0.14-0.49)	<0.001	0%	0.35 (0.14-0.88)	0.17	36%	0.22 (0.14-0.35)	<0.001	0%
CRP	−0.86 (−2.90-1.18)	0.41	55%	−1.97 (−5.39-1.45)	0.26	62%	NA	NA	NA	−1.47 (−3.29-0.35)	0.11	78%
KSS	1.79 (−0.84-4.43)	0.2	9%	2.10 (−0.30-4.50)	0.09	NA	NA	NA	NA	1.96 (0.19-3.73)	0.03	0%
HHS	2.77 (0.61-4.92)	0.012	26%	0.55 (−3.08-4.18)	0.77	NA	NA	NA	NA	2.19 (0.34-4.04)	0.021	22%
LoS (d)	−11.29 (−22.36-−0.22)	0.046	98%	6.81 (−0.02-13.64)	0.051	83%	NA	NA	NA	−2.46 (−13.91-8.99)	0.67	99%

CI = confidence interval, CRP = C-reactive protein, CSB = calcium sulfate bead, DAC = defensive antibacterial coating, HHS = Harris Hip Score, I^2^ = I-squared, KSS = Knee Society Score, LoS = length of hospital stay, NA = not applicable, OR = odds ratio

For functional outcomes, DAC showed the most favorable results. KSS improved modestly overall (MD = 1.96; 95% CI, 0.19 to 3.73; *P* = 0.03; I^2^ = 0%), whereas subgroup effects for DAC and CSB were directionally positive but individually notable. HHS demonstrated a notable improvement driven primarily by DAC (MD = 2.77; 95% CI, 0.61 to 4.92; *P* = 0.012; I^2^ = 26%), whereas CSB showed no notable benefit. Length of hospital stay (LoS) results were highly heterogeneous and inconclusive overall (MD = −2.46 days; *P* = 0.67; I^2^ = 99%); however, DAC was associated with a notable reduction in LoS (MD = −11.29 days; *P* = 0.046) in contrast to CSB, which showed a notable trend toward longer hospitalization.

Several outcomes were unavailable for BC and limited by single-study data in CSB, underscoring variability and data sparsity. The findings summarized in Table 3 suggest a pronounced decrease in infection risk and modest gains in functional outcomes—especially with DAC—whereas effects on systemic inflammatory parameters and length of hospital stay show considerable variability and lack consistent evidence.

Table 4 summarizes the Grading of Recommendations Assessment (GRADE) assessment for each outcome. Reinfection showed high-quality evidence with consistent and precise results. CRP was rated low due to moderate heterogeneity and wide CIs. KSS and HHS had moderate-quality evidence, downgraded for imprecision. LoS was rated very low due to substantial heterogeneity and imprecision.

**Table 4:** GRADE Analysis of Each Outcomes

Outcome	Study Design	Risk of Bias	Inconsistency	Indirectness	Imprecision	Publication Bias	Overall Quality
Infection rate	RCT, cohort, case-control	Low	Low (I^2^ = 0%)	No	No	No	High
CRP	RCT, cohort, case-control	Low	Moderate (I^2^ = 78%)	No	Yes (wide CI)	No	Low
KSS	RCT, cohort, case-control	Low	Low (I^2^ = 0%)	No	Yes (wide CI)	No	Moderate
HHS	RCT, cohort, case-control	Low	Moderate (I^2^ = 22%)	No	Yes (wide CI)	No	Moderate
LoS	Cohort and case-control	Low	High (I^2^ = 99%)	No	Yes (wide CI)	No	Very low

CI = confidence interval, CRP = C-reactive protein, HHS = Harris Hip Score, I^2^ = I-squared, KSS = Knee Society Score, LoS = length of hospital stay

### Meta-Regression

Meta-regression analyses for CRP outcomes (Table 5 and Figure 4) demonstrated that most study-level covariates did not markedly modify the treatment effect. Mean age and follow-up duration showed negative slopes, indicating a tendency toward greater CRP reduction with increasing age and longer follow-up; however, these associations were not statistically significant. By contrast, male proportion emerged as a notable moderator, with higher percentages of male participants associated with higher CRP MD (β = 0.112; 95% CI, 0.033 to 0.191; *P* = 0.005), suggesting less pronounced CRP reduction in predominantly male cohorts. The treatment subgroup (DAC versus CSB) did not markedly predict changes in CRP, as reflected by the substantial overlap of CI in the scatter plots. These results suggest that differences in demographic characteristics, rather than the type of intervention, may contribute to the observed between-study heterogeneity in CRP outcomes.

**Table 5:** Meta-Regression Analysis of Study-Level Predictors for CRP and Length of Hospital Stay Outcomes

Outcome Variable	Predictor	Coefficient	Standard Error	95% CI	*P* Value
CRP	Mean age	−0.184	0.148	−0.47-0.11	0.214
	Male percentage	0.112	0.04	0.03-0.19	0.005
	Mean follow-up duration	−0.105	0.1	−0.30-0.09	0.293
	DAC treatment	1.154	1.926	−2.62-4.93	0.549
LoS	Mean age	−1.806	2.394	−6.50-2.89	0.451
	Male percentage	0.972	0.726	−0.45-2.40	0.181
	Mean follow-up duration	−1.234	1.738	−4.64-2.17	0.478
	DAC treatment	−17.97	6.75	−31.20 to −4.74	0.008

CI = confidence interval, CRP = C-reactive protein, DAC = defensive antibacterial coating, LoS = length of hospital stay

**Figure 4:**
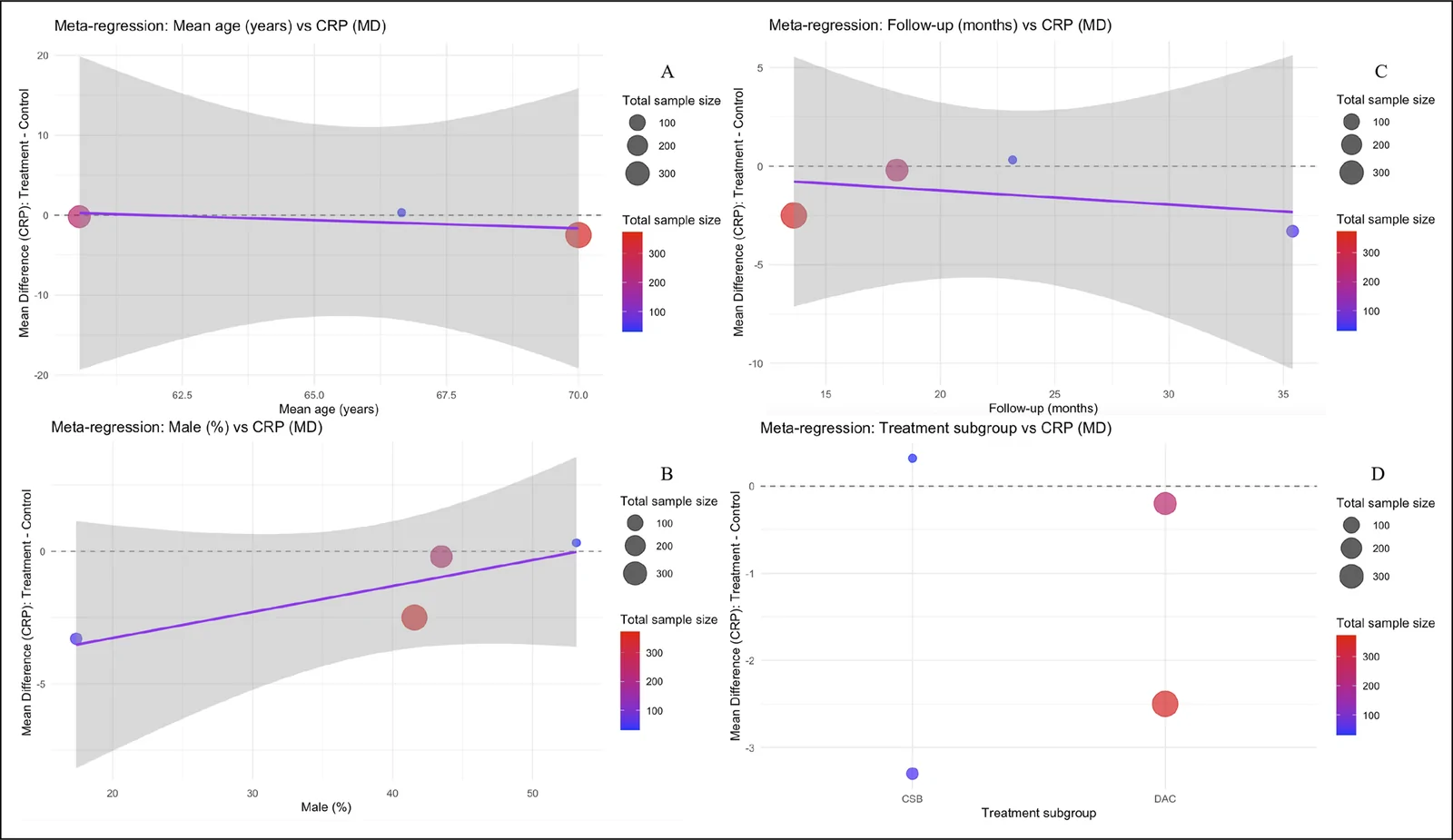
Scatter plots of meta-regression analyses examining study-level predictors of CRP outcomes. (**A**) Mean age, (**B**) male proportion, (**C**) follow-up duration, and (**D**) treatment subgroup (DAC versus CSBs). CRP = C-reactive protein, CSB = calcium sulfate bead, DAC = defensive antibacterial coating, MD = mean difference

For length of hospital stay (LoS; Table 5 and Figure 5), mean age, male proportion, and follow-up duration were not markedly associated with LoS differences, despite visually heterogeneous scatter patterns and wide CI, reflecting substantial residual heterogeneity. Treatment subgroup was a notable effect modifier, with DAC associated with a markedly greater reduction in LoS compared with CSB (β = −17.97 days; 95% CI, −31.20 to −4.74; *P* = 0.008). Collectively, the meta-regression results suggest that although most demographic and methodological factors do not consistently explain heterogeneity, male proportion influences CRP outcomes, and intervention type (DAC) is a key determinant of reduced LoS.

**Figure 5:**
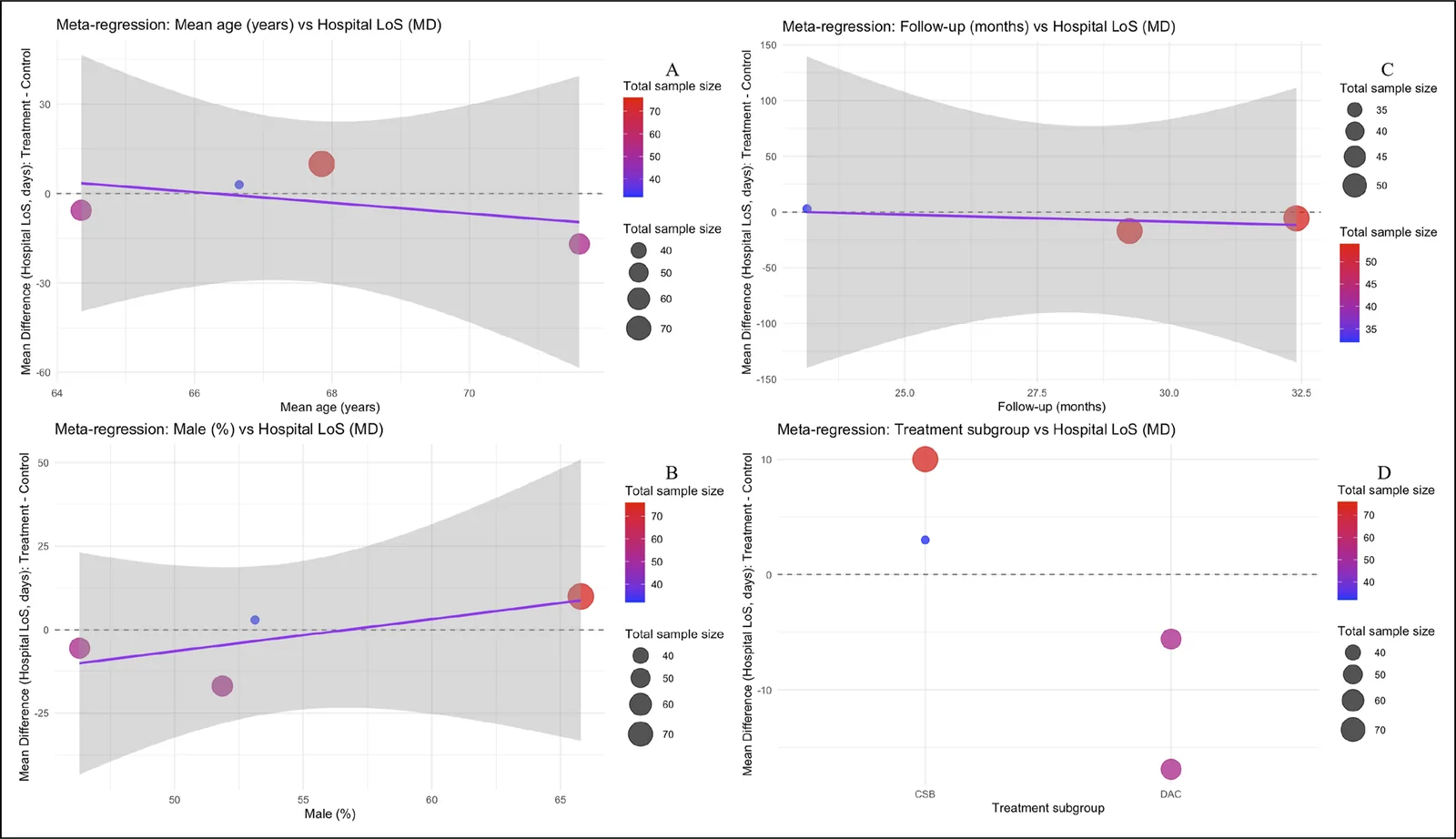
Scatter plots of meta-regression analyses examining study-level predictors of hospital LoS. (**A**) Mean age, (**B**) male proportion, (**C**) follow-up duration, and (**D**) treatment subgroup (DAC versus CSBs). CSB = calcium sulfate bead, DAC = defensive antibacterial coating, LoS = length of hospital stay, MD = mean difference

## Discussion

In this study, we found that DAC was associated with enhanced ability in reducing reinfection risk among the investigated local antibiotic regimens, with an OR of 0.14 (95% CI, 0.04 to 0.42), followed by BC and CSB. DAC also shortened the LoS by −11.29 days (95% CI, −22.36 to −0.22), and this may indicate a more refined surgical course with fewer infection-related problems. The functional outcomes of HHS and KSS between DAC and CSB remain inconsistent, as there was only one CSB study reporting HHS and KSS values. We were also unable to fully analyze the functional outcomes of BC due to a lack of reported data from the included studies. Overall, the present evidence suggests that DAC is associated with a notable reduction in reinfection rates, as indicated by the incidence rate and CRP as a marker.

The main advantage of effective local antibiotic therapy is that it may reduce the need for extensive systemic antibiotic therapy in certain patients. A good local antibiotic carrier is assessed based on its ability to deliver antibiotic over a prolonged duration at effective concentrations, as well as its capacity to degrade properly with minimal risk of allergy and side effects.^13^ The advantages and benefits of each antibiotic carrier are outlined in Table 6. DAC has been proposed as a prophylactic implant coating, as it transforms the implant surface from hydrophobic to hydrophilic to inhibit bacterial adhesion, yet is fully resorbed within 72 hours.^33^

**Table 6:** Advantages and Disadvantages of BC, CSBs, and DAC

Carrier Type	Advantages	Disadvantages
BC	PMMA provides a high local concentration of AB and is widely used in infection management due to its mechanical strength, which can support implants when used as BC. It is highly versatile and has been employed both for infection treatment and prevention in PJI cases	However, PMMA is nonbiodegradable and requires surgical removal after AB depletion, increasing patient morbidity. In addition, the exothermic polymerization process can degrade certain AB, potentially reducing their effectiveness. Another concern is that residual AB release over time may lead to subtherapeutic levels, increasing the risk of AB resistance
CSBs	CSB are fully resorbable, eliminating the need for removal, and can achieve high local AB concentrations without systemic toxicity. Their osteoconductive properties may also support bone healing, making them an attractive alternative to PMMA in PJI treatment	Despite these benefits, CSB has a shorter duration of AB release compared with PMMA, limiting its long-term effectiveness. In addition, some studies have reported transient hypercalcemia as a potential adverse effect. Because of their lack of mechanical strength, CSB cannot provide structural support, restricting their use in cases requiring load-bearing materials
DAC	DAC is a fully resorbable hydrogel that modifies implant surfaces, making them less conducive to bacterial adhesion while allowing for AB incorporation. Its biodegradability eliminates concerns about foreign material retention, and clinical studies have reported no major safety issues associated with its use	The primary limitation of DAC is its short duration of action, as it is completely resorbed within 72 hr, making it more suitable for infection prevention rather than long-term treatment. Furthermore, its effectiveness depends on direct application to the implant surface, restricting its use to intraoperative settings

BC = bone cement, CSB = calcium sulfate bead, DAC = defensive antibacterial coating, PJI = periprosthetic joint infection, PMMA = polymethylmethacrylate

A study by Zagra et al evaluated infection rates in patients with hip PJI undergoing a two-stage procedure using DAC-coated implants compared with uncoated implants. With an average follow-up of 2.7 ± 0.6 years across 27 cases, the study reported no infections in the DAC group,^40^ compared with four infections in the control group. Similarly, a study by Zoccali et al examined infection rates in 43 patients undergoing joint mega-prosthesis surgery with DAC-coated implants, compared with those without coating, with a follow-up period of up to 2 years. The results also demonstrated a lower infection rate in the DAC group (0%) versus the control group (13.9%). The reported reduction in infection rates related with DAC could be explained through a dual mechanism, a hyaluronic acid base that inhibits bacterial adhesion and the localized release of high-dose antibiotic. Its complete implant coverage and safe biodegradability further enhance its clinical utility.^42^

CRP, a widely used biomarker in the diagnosis and monitoring of PJI, were analyzed in this study, as it plays a crucial role in guiding treatment decisions and evaluating the success of infection eradication following THA or TKA.^56^ In a study by Malizos et al, consisting of 253 included patients undergoing osteosynthesis for a closed fracture, this study reported various of laboratory test results, one of it is CRP, which resulted a non–statistically significant result (4.2 ± 4.6 in the DAC group versus 4.1 ± 8.3 in the control group). Although DAC-coated implants markedly reduced surgical site infections, the CRP levels between the groups did not differ, this is likely due to the low overall infection rate and the timing of measurement at 6 months postoperatively, when most inflammatory responses had resolved. CRP, as a systemic marker, may not sensitively reflect localized, low-grade infection differences in such a context.^43^

As for the functional outcomes, a case-series study by Capuano et al, involving 27 patients with PJI undergoing a one-stage procedure using DAC-coated implants compared with 27 PJI patients undergoing two-stage revision using topical antibiotic, reported a HHS of 85.4 ± 3.6 in the DAC group versus 83.6 ± 7.4 in the two-stage revision group and KSS of 78.0 ± 6.1 in the DAC group versus 77.3 ± 6.4 in the two-stage group.^41^ In addition, a study by De Meo et al^34^ included 34 patients who underwent revision hip surgery, using a 1:1 matching method between those treated with an DAC and control patients undergoing osteosynthesis following a prior femoral neck fracture, with a minimum follow-up of 6 months. The HHS was markedly improved in the DAC group compared with the control group. These findings are consistent with our results, showing a notable improvement in HHS and a slight, but not statistically significant, improvement in KSS. Conversely, the lack of statistical significance in KSS between DAC and control groups may be due to the low overall infection rate, small sample size in the knee subgroup, and the multifactorial nature of postoperative knee recovery, which may have obscured the functional benefits of infection control in the short term.^41^

A study by Zagra et al^40^ revealed that patients in the DAC group were associated with lesser LoS in comparison to the control group (28.2 ± 3.9 vs. 33.8 ± 5.4 days). In addition, a study by Capuano et al,^41^ reported DAC group was also associated with a shorter LoS (18.9 ± 2.9 vs. 35.8 ± 3.4). In this study, the markedly shorter hospital stay in the DAC group is most likely caused by a lower frequency of surgical infections, which may lessen the need for extended antibiotic therapy and reintervention. In addition, the safe, resorbable nature of DAC supported smoother recovery and simplified postoperative care, enabling earlier mobilization and discharge.^57^

Besides DAC, CSB were also evaluated as a local antibiotic carrier. In a study by Tarity et al, which included 40 patients, the addition of CSB to the DAIR procedure did not reduce infection rates compared with standard DAIR during a 90-day follow-up. This is likely due to the limited utility of CSB in acute PJI, where critical factors such as biofilm presence, pathogen virulence, and the extent of modular component exchange play a larger role in treatment outcomes.^49^ Similarly, Piovan et al conducted a retrospective cohort study involving 32 patients, which showed comparable success rates between DAIR alone (80%) and DAIR with CSB (88%) but no notable differences in infection eradication. The study also examined potential CSB-related adverse events, including hypercalcemia, heterotopic ossification, and wound drainage, but no such complications were observed.^44^

Dimofte et al further evaluated CSB use in a cohort of 76 patients with a mean follow-up of 51 months. They found that patients in the CSB group experienced a longer hospital stay compared with those receiving standard DAIR (22 vs. 13 days). This prolonged hospitalization is likely multifactorial, potentially due to the need for closer postoperative monitoring, inflammatory responses leading to prolonged wound drainage, and higher patient complexity prompting more cautious clinical management.^48^ Although CSB presents a viable alternative for local antibiotic delivery, current evidence suggests that it does not offer superior outcomes when compared with DAC.

CSBs are frequently used in DAIR surgeries as a local antibiotic delivery system, capable of releasing high concentrations of antibiotic directly at the surgical site to enhance infection control.^48^ A study by McPherson et al investigated the use of CSB in both aseptic revisions and second-stage reimplantation THA. In this retrospective cohort study involving 215 patients, the intervention group received point-of-care application of antibiotic -loaded CSB, whereas the control group underwent standard procedures. With a mean follow-up of 56.6 months in the intervention cohort, the study found no statistically significant difference in PJI rates for aseptic revisions, both groups reported a rate of 1.4%. However, in the second-stage reimplantation subgroup, the PJI rate was markedly lower in the intervention group (4.8%) compared with the control group (23.3%).^19^

Two-stage revision surgery has long been considered the benchmark due to its historically high success rates in managing PJI.^58^ However, two-stage revision is associated with prolonged treatment duration, increased surgical trauma, longer hospitalization, and higher costs, which can negatively affect patient recovery and satisfaction. By contrast, single-stage revision surgery, which entails removal of the infected prosthesis and immediate reimplantation during the same surgery, has gained increasing attention worldwide for its potential to reduce surgical morbidity, shorten hospital stay, lower overall costs, and improve functional recovery without compromising infection control.^59^

A case series by Pellegrini and Legnani is the first to introduce the use of DAC in single-stage revision surgery of THA. After an average follow-up period of 3.1 years, the study found that 10 patients experienced notable improvements in functional outcomes, with the HHS increasing from a preoperative mean of 38.1 ± 10.3 to 81.3 ± 6.7 (*P* < 0.001), and visual analog scale (VAS) pain scores decreasing from 6.8 ± 3.2 to 1.9 ± 2.4 (*P* < 0.001).^35^ An ongoing retrospective matched cohort study, synthetic injectable bone substitute–hydrogel (SINBOSE-H), by Boyer et al^39^ is also aiming to prove that single-stage surgery with AB-loaded DAC is non-inferior to two-stage surgery of chronic hip PJI treatment. These studies support the growing interest in evaluating single-stage revision as a potential alternative for managing PJI.

Besides DAC, several other carriers are used to enhance antibiotic delivery and biofilm eradication. Such as dual antibiotic regimen, prolonged perioperative and postoperative antibiotic prophylaxis, and nanocarrieers and bacteriophage. The dual antibiotic regimen involves the simultaneous use of two antibiotic typically one systemic (IV or oral) and one locally delivered (eg, in spacers or coatings) to broaden antimicrobial coverage and enhance bacterial killing, particularly against resistant strains. Although this approach offers improved infection control and broader spectrum activity, it also increases the risk of side effects, treatment burden, and requires close monitoring and patient adherence.^60^

Prolonged perioperative and postoperative antibiotic prophylaxis refers to the extended administration of systemic antibiotic beyond the standard 24 to 48 hours after surgery, often continuing for several days to weeks. This strategy aims to reduce the risk of infection, especially in high-risk or revision cases such as those involving PJI, although it may also raise concerns about antibiotic resistance and adverse effects.^61^ Other currently ongoing trials are the nanocarriers and bacteriophages. Although both bacteriophages and nanocarriers offer promising advantages—such as targeted action, biofilm penetration, and reduced systemic toxicity, their clinical application is currently limited by regulatory challenges, production complexity, potential immune responses, and high development costs.^8,62^

This systematic review and meta-analysis offers several notable strengths. A comprehensive literature search across five major databases enhanced the completeness and reliability of the evidence synthesis. By including randomized controlled trials, cohort studies, and matched case-control studies, the analysis captures a broad spectrum of real-world clinical scenarios involving total hip and knee arthroplasty. The use of validated meta-analytic methods in RStudio, along with the application of the GRADE framework to assess evidence quality, strengthens the validity and transparency of the findings. The inclusion of diverse and clinically meaningful outcomes—such as infection rate, CRP levels, HHS, KSS, and length of hospital stay—provides a well-rounded comparison of the effectiveness of local antibiotic carriers. Among the interventions analyzed, DAC was associated with favorable outcomes across several clinically relevant endpoints, supporting its potential clinical advantage.

However, the study has several limitations that should be considered. Many of the included studies were observational in nature, making the findings susceptible to selection bias and residual confounding. For several outcomes, particularly CRP and KSS, the pooled effects did not reach statistical significance, potentially reflecting limited statistical power or heterogeneity in outcome measurement. Differences in the types and dosages of antibiotics used across studies further introduce clinical heterogeneity that could not be fully controlled. In addition, the review was restricted to English-language articles, which may have led to language bias and the exclusion of relevant studies. Some unpublished or grey literature might also have been missed. These limitations suggest that although DAC appears promising, high-quality randomized controlled trials directly comparing DAC, CSB, and BC with standardized protocols are warranted. The findings underscore the potential of DAC in reducing PJI, and future research should also address cost-effectiveness and long-term safety to guide clinical policy and practice.

## Conclusions

Among the evaluated local antibiotic delivery systems, DAC was associated with more favorable trends in infection prevention, length of hospital stay, and functional outcomes following total hip and knee arthroplasty. However, these findings should be interpreted with caution, as the available evidence is derived largely from observational studies, involves a limited number of DAC-specific investigations, and includes heterogeneous patient populations without robust head-to-head comparisons between delivery systems. Although DAC demonstrated notable reductions in infection-related outcomes and modest improvements in functional scores in some analyses, CSB and BC also showed potential benefits in infection control, albeit with more variable and frequently negligible effects on inflammatory markers, function, and hospitalization duration. Overall, the current evidence suggests that DAC may represent a promising local antibiotic delivery strategy, but definitive conclusions regarding its superiority over established methods cannot yet be drawn. Well-designed, adequately powered, randomized, controlled trials directly comparing local antibiotic delivery systems are needed to clarify their relative effectiveness and inform routine arthroplasty practice.
